# Flexible Sigmoidoscopy Utility in the Diagnosis of Pediatric Gastrointestinal Disorders

**DOI:** 10.7759/cureus.38553

**Published:** 2023-05-04

**Authors:** Catalina Jaramillo, Anna K Ermarth, John S Collier, John F Pohl, Raza A Patel

**Affiliations:** 1 Pediatric Gastroenterology, University of Utah Health, Salt Lake City, USA

**Keywords:** diagnostic, colitis, gastrointestinal, pediatric, sigmoidoscopy, flexible

## Abstract

Aim: Although flexible sigmoidoscopy (FS) is utilized in children for the diagnosis of pediatric gastrointestinal conditions, such as inflammatory bowel disease and juvenile polyp disorders, the diagnostic yield of FS in pediatric patients is unknown.

Materials and methods: We retrospectively reviewed FS cases in children under 18 years of age over a five-year period at our institution. Indications for the procedure, endoscopic visual findings, histologic findings, final diagnosis, and any management changes based on FS findings were included.

Results: A total of 354 cases were included in the analysis for which 40 cases (11.3%) had abnormal visual findings, 48 cases (13.6%) had abnormal histologic findings, and 13 cases (3.7%) had both abnormal endoscopic visual and histologic findings. Of the 88 cases with abnormal visual and/or histologic abnormalities, only the results of 34 of these FS cases led to a change in management based on endoscopic findings (9.6%). Most patients with a non-diagnostic FS had a final diagnosis of functional abdominal pain; most patients with a diagnostic FS had a final diagnosis of colitis, not otherwise specified.

Conclusion: Our findings suggest that FS is not a helpful diagnostic endoscopic intervention in pediatric patients, especially in children with reassuring history and physical exam findings.

## Introduction

Flexible sigmoidoscopy (FS) has been validated as an important tool for colorectal cancer screening in adults [1,2]. However, in pediatric patients, its value as a diagnostic procedure is not clear. Indications cited for diagnostic FS often overlap with those listed for colonoscopy, including abdominal pain, diarrhea, and rectal bleeding. While successful colonoscopy requires complete bowel preparation, including ingesting a large volume of osmotic laxatives, which is difficult for some children to tolerate, preparation for FS requires smaller-volume laxatives and/or enema administration. In clinical practice, this difference may cause both clinicians and parents to consider a potentially less-invasive FS evaluation for some indications, such as an investigation for histologic evidence of graft-versus-host disease (GVHD) [3,4]. In many situations (e.g., in patients with non-specific lower gastrointestinal symptoms), the decision to perform FS as an alternative to colonoscopy in a pediatric patient is not clear. Such a decision can be problematic as prior clinical research has suggested that the findings of normal mucosa via colonoscopy have a high correlation with normal colonic tissue biopsy findings in children with abdominal pain [5]. To date, there have been no studies designed specifically to determine the diagnostic yield of FS in pediatric patients, and this retrospective cohort study seeks to narrow this gap.

## Materials and methods

We performed a retrospective review of de-identified patients under 18 years of age who underwent FS between January 2016 and March 2021 identified by the Intermountain Children’s Health Pediatric Analytics with the approval of both Primary Children’s Hospital and the University of Utah Institutional Review Boards (IRB 00138536). For all FS cases, the following information was captured by chart review: age (years) at the time of the FS, sex, date of the procedure, indications for the procedure, endoscopic visual findings as documented in the procedure note, histologic findings as detailed in the pathology report, the final diagnosis as documented by the patient’s primary gastroenterologist, and any changes in management that were prompted based on the findings of FS. Cases that were performed in conjunction with esophagogastroduodenoscopy (EGD) also were noted. Exclusion criteria included conversion from colonoscopy to FS due to poor bowel preparation or severe mucosal disease preventing the advancement of the colonoscope, a known mucosal disease noted from previous FS, repeat FS performed on the same patient, FS performed with no biopsies taken, and suspected GVHD cases.

Visual findings were classified as “normal” (i.e., unremarkable-appearing colonic mucosa) or “abnormal” per the procedure reports if the colonic mucosa had any mucosal changes. Histologic findings also were classified as normal or abnormal based on pathology reports. Lymphonodular hyperplasia (LNH) was designated as a normal histologic finding as LNH is considered a benign, asymptomatic finding in most pediatric patients [6]. A “diagnostic” FS was defined as a procedure that yielded abnormal visual and/or histologic findings leading to a specific diagnosis and resulting in a change in the patient’s management. Changes in management included initiation of medication, initiation of dietary therapy, or use of an intervention (such as a polypectomy). All other FS cases were considered “non-diagnostic.” The recommendation to initiate or escalate therapy for constipation was not considered to be a change in management prompted by the findings of FS, as the diagnosis of functional constipation can be made clinically.

Patient characteristics were summarized using descriptive statistics. Categorical data was analyzed using two-sample Wilcoxon-Mann-Whitney rank-sum and Pearson’s chi-squared tests where appropriate. Significance between groups was defined as a p-value < 0.05 throughout. The above analyses were performed using Stata-13 statistical software (College Station, TX, StataCorp LP).

## Results

**Figure 1:**
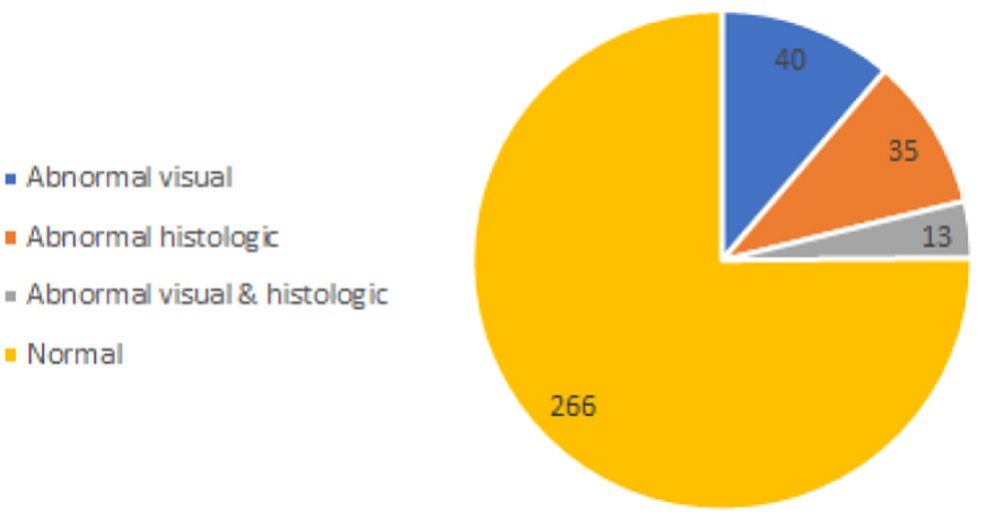
Findings of all FS cases (total 354)

A total of 445 eligible patient FS reports were identified from both the inpatient and outpatient settings, and a concurrent EGD occurred in 92% of FS cases. Using exclusion criteria as defined above, 91 cases were removed from the analysis. As shown in Figure 1, 40 cases (11.3%) of the remaining 354 FS cases had abnormal visual findings.

It was noted that 48 cases (13.6%) had abnormal histologic findings, and within these, 13 cases (3.7%) also had abnormal endoscopic visual findings. Of these 88 cases with abnormal visual and/or histologic abnormalities noted, 34 of these FS cases were considered diagnostic in that those patients underwent a change in management based on their FS results (9.6% of total FS cases). Of note, there was no significant difference in age and sex between patients with normal FS and abnormal FS findings. No data was available regarding the depth of insertion of the endoscope from the anal verge. No FS procedure was performed on any patient with a history of intestinal transplantation.

The most common indications for performing an FS with diagnostic findings were diarrhea (n=20 patients) and abdominal pain (n=11 patients), followed by smaller numbers of patients in descending order with rectal bleeding, eosinophilic esophagitis (EoE)/dysphagia, vomiting, dyschezia/rectal pain, and malnutrition. The most common clinical indications for performing an FS in patients with non-diagnostic findings were diarrhea (n=140 patients) and abdominal pain (n=135 patients), followed by a smaller number of patients in descending order with vomiting, rectal bleeding, dyschezia/rectal pain, EoE/dysphagia, constipation, and malnutrition. Some patients had more than one of these diagnoses at the time of FS. For example, some patients with a diagnosis of EoE/dysphagia or vomiting underwent FS due to concerns of concurrent eosinophilic gastrointestinal disease [7].

Table 1 demonstrates that most patients with an abnormal FS (by visualization or biopsy) had a final diagnosis of colitis that was not otherwise specified (42% of diagnostic FS cases), followed by eosinophilic colitis (30% of diagnostic FS), colonic polyp (9%), Crohn's disease (6%), Enterobius infection (6%), collagenous colitis (3%), and solitary rectal ulcer syndrome (3%).

**Table 1:** Abnormal visual and histological FS findings in 88 patients

Diagnosis	Number	Percentage
Colitis not otherwise specified	37	42
Eosinophilic colitis	27	30
Polyp	8	9
Crohn's disease	5	6
Enterobius infection	5	6
Collagenous colitis	3	3
Solitary rectal ulcer	3	3

Some patients had more than one diagnosis after a diagnostic FS was performed. The final diagnosis of the 34 FS cases leading to a change in treatment consisted of eosinophilic colitis (nine patients), colitis not otherwise specified (eight patients), other diagnoses (seven patients), Crohn's disease (four patients), unspecified diagnosis (two patients), polyp (two patients), functional constipation (one patient), and collagenous colitis (one patient). Most patients with a normal FS (by visualization or biopsy) had a final diagnosis of functional abdominal pain (n=74) and functional constipation (n=63), followed by smaller numbers of patients in descending order with disaccharidase deficiency (if an EGD was performed at the time of FS), EoE (if an EGD was performed at the time of FS), and non-specific diarrhea of childhood which did not change the initial diagnosis or management.

## Discussion

In our retrospective study, only 9.6% of the 354 pediatric FS procedures were diagnostic and resulted in a change in the patient’s management. Our results demonstrate that FS is not helpful as a diagnostic tool if a pediatric patient is having symptoms that clinically match disorders such as functional abdominal pain. Data from other studies have suggested that pediatric colonoscopy is a safe procedure that should be performed if gastrointestinal disease with pathologic colonic findings is strongly being considered, and the national PEDS-CORI (Pediatric Endoscopy Database System-Clinical Outcomes Research Initiative) database has shown that pediatric colonoscopy appears to be a more common procedure compared to FS [8,9]. In our study, the most common diagnosis determined based on abnormal visual ± diagnostic findings on FS was colitis (not otherwise specified) and eosinophilic colitis. In such a setting, a colonoscopy might have been a better diagnostic option and could have changed treatment management considerably if such patients had overlapped with other disorders such as Crohn's disease with ileal involvement [10,11]. Additionally, the presence of a polyp found by FS does not exclude the presence of polyps of the distal colon which would be found by colonoscopy [12]. However, FS may be helpful and safe in specific conditions such as intestinal failure or GVHD [3-4,13]. Prior research has demonstrated that pediatric gastroenterologists often will decide between a diagnostic FS versus a colonoscopy in the setting of needing to know the disease location, regardless of procedural cost [14]. Thus, gastrointestinal diseases with diffuse pathology (such as GVHD) might benefit from a relatively simple FS compared to a colonoscopy.

In our study, patients with an ultimate diagnostic FS had visual ± histologic changes compared to patients with a non-diagnostic FS which inherently makes sense. However, the high rate of non-diagnostic FS in this retrospective study suggests that accurate history and physical exam findings for functional abdominal pain and chronic constipation would eliminate the need for FS in the first place, especially in the setting of the cost associated with FS (up to $3000 USD) while not accounting for further costs associated with biopsy processing, pathologist interpretation, and anesthesia [15]. Additionally, the percentage of finding diagnostic abnormalities in pediatric patients using FS in our study is similar to the percentage of finding diagnostic abnormalities in pediatric patients with functional abdominal pain undergoing EGD in one study while also being much lower compared to another study utilizing EGD in pediatric patients with chronic abdominal pain [16,17].

## Conclusions

In summary, our retrospective study suggests that most patients who underwent an FS at our institution did not have diagnostic findings that would have led to a change in treatment. Our results suggest that a reassuring history and physical examination for relatively benign gastrointestinal symptoms (such as functional abdominal pain or chronic constipation) should alleviate patient, family, and physician concern for organic disease while reducing the need for unnecessary FS evaluation. Weaknesses in this study consisted of having no data on this patient group in regards to testing for fecal biomarkers of inflammation such as lactoferrin and calprotectin as well as having no data on how many patients may have eventually needed a colonoscopy after an initial FS. Additionally, some patients in this retrospective study had a final diagnosis of "other diagnosis" and "unspecified diagnosis" due to a lack of a clear final diagnosis in the medical record leading to a change in therapy. More research is needed to determine if the use of FS has specific indications (compared to colonoscopy) for specific pediatric gastrointestinal disorders.
